# Effect and accuracy of emergency dispatch telephone guidance to bystanders in trauma: post-hoc analysis of a prospective observational study

**DOI:** 10.1186/s13049-016-0343-z

**Published:** 2017-03-07

**Authors:** Håkon Kvåle Bakke, Tine Steinvik, Håkon Ruud, Torben Wisborg

**Affiliations:** 1 Mo i Rana Hospital, Helgeland Hospital Trust, Mo i Rana, Norway; 2 Anaesthesia and Critical Care Research Group, Faculty of Health Sciences, IKM, University of Tromsø, Tromsø, Norway; 3 University Hospital of Northern Norway, Department of Emergency and Acute Care, Harstad, Norway; 4 Hammerfest Hospital, Department of Anaesthesiology and Intensive Care, Finnmark Health Trust, Hammerfest, Norway; 5 Norwegian National Advisory Unit on Trauma, Division of Emergencies and Critical Care, Oslo University Hospital, Oslo, Norway

**Keywords:** Trauma, First aid, Dispatch

## Abstract

**Background:**

Emergency medical communication centres (EMCCs) dispatch and allocate ambulance resources, and provide first-aid guidance to on-scene bystanders. We aimed to 1) evaluate whether dispatcher guidance improved bystander first aid in trauma, and 2) to evaluate whether dispatchers and on-scene emergency medical services (EMS) crews identified the same first aid measures as indicated.

**Methods:**

For 18 months, the crew on the first EMS crew responding to trauma calls used a standard form to assess bystander first aid. Audio recordings of the corresponding telephone calls from bystanders to the EMCC were reviewed.

**Results:**

A total of 311 trauma calls were included. The on-scene EMS crew identified needs for the following first-aid measures: free airway in 26 patients, CPR in 6 patients, and hypothermia prevention in 179 patients. EMCC dispatchers advised these measures, respectively, in 16 (62%), 5 (83%), and 54 (30%) of these cases. Dispatcher guidance was not correlated with correctly performed bystander first aid. For potentially life saving first aid measures, all (20/20) callers who received dispatcher guidance attempted first aid, while only some few (4/22) of the callers who did not receive dispatcher guidance did not attempt first aid.

**Discussion:**

Overall, the EMCC dispatchers had low sensitivity and specificity for correctly identifying trauma patients requiring first-aid measures. Dispatcher guidance did not significantly influence whether on-scene bystander first aid was performed correctly or attempted in this study setting, with a remarkably high willingness to perform first-aid. However, the findings for potentially lifesaving measures suggests that there may be differences that this study was unable to detect.

**Conclusion:**

This study found a high rate of first-aid willingness and performance, even without dispatcher prompting, and a low precision in dispatcher advice. This underlines the need for further knowledge about how to increase EMCC dispatchers’ possibility to identify trauma patients in need of first aid. The correlation between EMCC-guidance and bystander first aid should be investigated in study settings with lower spontaneous first-aid rates.

## Background

In trauma cases, bystander first aid can improve survival [1]. Emergency medical communication centres (EMCCs) dispatch ambulances and provide guidance to bystanders. Dispatcher guidance reportedly increases the bystander CPR rate following out-of-hospital cardiac arrest (OHCA) [2, 3]. However, dispatchers may have difficulty correctly assessing situations, [3, 4] and no studies have described bystander first-aid guidance in trauma cases.

We have previously published a study assessing which first aid measures bystanders performed in trauma. In that study we found a high rate of first aid intervention from bystanders, securing airway and recovery position were done in 76% of patients where those measures were indicated, bleeding control was done in 81%, hypothermia prevention for 62% of patients where needed [5]. Assessment of EMCC dispatch in OHCA has led to improved dispatch protocols and EMCC guidance. Therefore we wanted to make an assessment of EMCC guidance in trauma by reviewing the EMCC dispatch audio recordings for the EMS calls of our previous study. As the study was a follow-up on already prospectively collected first aid assessments no sample size calculation was performed.

Aims: 1) evaluate whether dispatcher guidance improved bystander first aid in trauma, and 2) evaluate whether dispatchers and on-scene emergency medical services (EMS) personnel identified similar first-aid needs.

## Methods

We performed a prospective observational study in the two northernmost health trust regions of Norway. Between October 2012 and April 2014, at the scene of each immediate-response trauma call (ICD-10 categories V01–Y98, excluding intoxications), the first on-scene EMS unit evaluated the first aid rendered prior to their arrival in a standard form. This included whether first aid measures open airway, recovery position, bleeding control, CPR, and hypothermia prevention were indicated and whether they were performed correctly or not at all. Measures were considered to be performed correctly if they were in accordance with EMS standard operating procedure for basic life support (BLS). Further details regarding the methods and findings concerning on-scene bystander first aid are reported elsewhere [5]. Audio recordings of the corresponding telephone calls from bystanders to the EMCC were identified and reviewed (by TS) using a data extraction form.

First-aid indications were determined according to the on-scene assessment by EMS. Free airway and recovery position were considered indicated in patients with anamnestic loss of consciousness, or GCS of <13 upon EMS arrival. The first aid measure bleeding control was not included, as the previous study did not differentiate between different severities of bleeding [5]. Sensitivity and specificity of the EMCC for identifying indicated first aid measures were analysed per measure, and one patient could have guidance given or indication for more than one first aid measure. When investigating the association between EMCC guidance and on-scene first aid, first aid measures that had been performed prior to telephone contact with the EMCC were excluded from analysis.

Norwegian EMCC operators use a criteria-based dispatch (CBD) system (Index) suggesting appropriate first-aid measures based on caller descriptions of signs and symptoms at the operator’s discretion [6]. We compared the performance of this use of the dispatch system to performance if the Index had been used as a strict algorithm. Statistical analyses were performed using SPSS Statistics for Mac version 21 (IBM Corp., Armonk, NY, USA).

The study was approved by the Regional Committee for Medical and Health Research Ethics, University of Tromsø (Ref. 2010/3328/REK nord).

## Results

Among 408 eligible cases, 355 EMCC audio recordings were retrieved, 73 of which were shared by multiple patients. Thus, 311 trauma calls were included in our analysis. Callers were usually members of the general public (Table 1), and were alone with the patient in 16% of cases (49/311). In 57% of (177/311) calls, the caller performed on-scene first aid.

**Table 1:** Identity of the person calling the emergency medical communication centre for 311 emergency calls

Caller identity	n	%
General public	170	55
Next of kin	49	16
On-duty healthcare personnel	34	11
Police officer on duty	31	10
Fire fighter on duty	4	1
Patient	23	7
Total	311	100

The EMCC dispatcher did correctly identify indicated first aid measures for 35% of all indicated measures. Table 2 presents the sensitivity and specificity in the EMCC for each measure. Using the Index as a strict algorithm would have led to a 94% sensitivity (95% CI 89–96) and 8% specificity (95% CI 6–10) for all measures combined. To identify factors that were associated with failed EMCC identification of indicated measure logistic regression analyses were performed. Cause of injury, identity of caller (as per Table 1), multiple patients, and whether the caller was also the person giving first aid were investigated. Analyses were first conducted for all measures, and then analyses separately for the life saving measures free airway, recovery position, and CPR. None of the factors were associated with failed EMCC identification of indicated measure, overall (Binary logistic regression, *p* = 0.64) or for potentially life saving measures (Binary logistic regression, *p* = 0.09).

**Table 2:** Analysis of the sensitivity and specificity in first responder guidance by the emergency medical coordination centre of relevant first aid measures in 311 trauma cases (Each patient could have guidance given or indication for more than one first-aid measure)

First-aid measure	EMS assessment	EMCC guidance given	EMCC guidance not given	EMCC sensitivity & specificity (95% CI)
Free airway				
	Indicated (26)	16	10	62% (41–79)
	Not indicated (285)	10	275	96% (93–99)
CPR				
	Indicated (6)	5	1	83% (36–99)
	Not indicated (305)	4	301	99% (96–99)
Recovery position				
	Indicated (23)	7	16	30% (14–53)
	Not indicated (288)	9	279	97% (94–98)
Hypothermia prevention				
	Indicated (179)	54	125	30% (24–38)
	Not indicated (132)	6	126	95% (90–98)
Total				
	Indicated (234)	82	152	35% (29–42)
	Not indicated (1010)	29	981	97% (96–98)

*EMS* emergency medical services, *EMCC* emergency medical communication centre

**Fig. 1:**
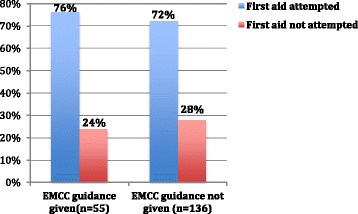
First-aid measure attempts with and without EMCC guidance. There was no statistically significant difference in first aid attempts between bystanders receiving EMCC guidance and those not receiving EMCC guidance (*p* = 0.592)

EMCC guidance was not associated with correct first-aid performance, overall or for any single first aid measure (Chi-square tests, *p* = 0.3–0.6). Neither was EMCC guidance associated with whether bystander first aid was attempted (Chi-square tests, *p* = 0.11–0.6; Fig. 1). The potentially life-saving measures free airway, recovery position, and CPR, where then analysed separately. First aid had been attempted for all cases (*n* = 20) when EMCC guidance was given and one of these measures was indicated. When EMCC guidance had not been given 4/22 did not attempt first aid for these measures when indicated (Chi-square test, *p* = 0.109).

## Discussion

We found that EMCC guidance to bystanders did not affect how often first aid was attempted, or first-aid quality, in this sample with high spontaneous bystander first aid provision. In addition, EMCC dispatchers had a low sensitivity for identifying indicated first-aid measures.

In OHCA there is conflicting evidence regarding the effect of EMCC guidance on survival, but such guidance has been shown to considerably improve the rate of bystander CPR [2]. Our study showed no significant effect of EMCC guidance to bystanders on rate of first aid. This may be because bystander first aid rates in the study area were generally high, and EMCC guidance therefore has little additional effect. Another possible interpretation is a more modest effect on bystander first aid of guidance in trauma compared to OHCA. On the other hand, for the life saving measures, all bystanders who received EMCC guidance had performed the indicated first aid measures, whereas some of those who did not receive guidance had not performed necessary measures. The cohort under study also had relatively few severely injured patients. It is therefore conceivable that EMCC guidance actually have effect on rate of bystander first aid, but that further studies with larger sample sizes are needed.

Prior studies of OHCA report correct cardiac arrest diagnosis in 15–92% of cases, [3, 4] the overall sensitivity in our study was in the lower range of this at 35%.

From OHCA studies has shown that EMCC sensitivity can be increased through improved protocols [3]. Our data indicated that strict adherence to the Index could improve EMCC sensitivity, but with greatly reduced specificity. This assumes however that the on-scene situation is easily discernible for the EMCC dispatcher and that the Index is easy to apply where several problems are present on-scene. Moreover first-aid guidance by telephone is time consuming, and a wholly scripted approach where all first aid measures were instructed is not likely to be effective. Indeed, comparison between a system strictly scripted EMCC guidance and a system where more decisions are left to the dispatchers’ discretion showed that CPR-instructions where offered faster and more frequently in the latter [6].

Improved guidance of first responders by EMCC’s relies on better identification of patients in need. This study failed to identify clues in cause of injury, identity of caller, number of victims or the callers’ actual involvement in providing first aid to influence the precision of the recognition of situations needing guidance. However, first aid measures were relatively seldom indicated in the study population, and similar analyses should be conducted in larger studies.

This study is limited by the small cohort, and relatively few severely injured patients. The decision to review the sound logs for the EMCC guidance was done retrospectively. The results should be interpreted cautiously, and are mainly useful for hypothesis generation. This cohort showed a high rate of bystander-initiated first aid in trauma, which may limit the impact of EMCC guidance.

## Conclusions

EMCCs have difficulty correctly identifying trauma patients requiring several first-aid measures. EMCC dispatcher guidance did not seem to affect whether on-scene bystander first aid was performed correctly or attempted., in an area with a high rate of bystander first aid.

## Abbreviations

BLS: Basic life support
CPR: Cardiopulmonary resuscitation
EMCC: Emergency medical communication centre
EMS: Emergency medical services
OHCA: Out-of-hospital cardiac arrest
